# The urgent need for newer drugs in routine HIV treatment in Africa: the case of Ghana

**DOI:** 10.3389/fepid.2025.1523109

**Published:** 2025-03-14

**Authors:** Sekyibea Nana Ama Appiedu-Addo, Mark Appeaning, Edwin Magomere, Gloria Akosua Ansa, Evelyn Yayra Bonney, Peter Kojo Quashie

**Affiliations:** ^1^ West African Centre for Cell Biology of Infectious Pathogens (WACCBIP), College of Basic and Applied Sciences, University of Ghana, Accra, Ghana; ^2^ Department of Biochemistry Cell and Molecular Biology, School of Biological Sciences, College of Basic and Applied Sciences, University of Ghana, Accra, Ghana; ^3^ Medical Laboratory Sciences Department, Koforidua Technical University, Koforidua, Ghana; ^4^ Public Health Department, The Bank Hospital, Accra, Ghana; ^5^ Virology Department, Noguchi Memorial Institute for Medical Research (NMIMR), College of Health Sciences, University of Ghana, Accra, Ghana; ^6^ The Francis Crick Institute, London, United Kingdom

**Keywords:** ART, HIV/AIDS, Africa, new drugs, treatment

## Abstract

Antiretroviral therapy (ART) has tremendously improved the quality of life of people living with HIV (PLWH). Through rigorous scientific research and development, newer, more effective, and less toxic antiretrovirals (ARVs) have been developed and are available to PLWH in high-income countries (HICs). Although Africa accounts for more than two-thirds of the global burden of HIV/AIDS, this large population does not readily have access to these newer and more effective ARVs. In some instances, new ARVs become available to PLWH in Africa over a decade after they have been approved for use by the Food and Drug Authorities (FDAs) in HICs. Since 2010, 35 new drug entities have been approved; of those, only 3 are in common use in Ghana and most of Sub-Saharan Africa. To achieve the 2030 goal of ending HIV/AIDS as a global health epidemic, it is critical to ensure equity in access to newer and effective ARVs across all regions, including Africa, where the majority of PLWH reside. We highlight here the urgent need to make newer ARVs available in Africa to ensure the realization of the Global End AIDS by 2030 goal.

## Background

By the end of 2023, an estimated 39.9 million people were living with HIV/AIDS globally, with 630,000 HIV-related deaths recorded that year (1). Africa accounted for approximately 70% of all HIV cases, contributing significantly to the global burden, including 390,000 HIV-associated deaths. Eastern and Southern Africa bore the highest burden, with 20.5 million people living with HIV (PLWH), while Western and Central Africa had an estimated 5.5 million PLWH at the same time (1). Despite a 50% decline in HIV-related deaths across Africa from 2010 to 2023 (decreasing from 890,000 to 390,000), mortality rates in the region remain disproportionately high compared to the global average (1). These figures underscore the need for intensified efforts to reduce the burden of HIV/AIDS in Africa.

The current global HIV goal is to end HIV/AIDS as a public health threat by 2030 (2). The world missed the 2020 target of 90–90–90 [90% of all people living with HIV being aware of their status, 90% of those diagnosed receiving antiretroviral therapy (ART), and 90% of those receiving ART achieve viral suppression], it is essential that the most affected regions including Africa is not left behind in the current targets (3). Despite the gains made with Antiretroviral (ARV) development (Table 1), it will be challenging to reduce the HIV burden if more effective antiretrovirals (ARVs) are not made available in the region. HIV transmission remains high, with approximately 1.1 million people being newly infected annually in Africa despite the expanded access to ART (4). A longitudinal study in South Africa revealed an increase in HIV incidence, within the cohort, from 21% to 29% from 2004 to 2011, during a period when ART coverage for all PLWH was introduced (5). Thus, despite the scale-up of access to ART, transmission remains high, raising doubts about the efficacy of the ARVs used in low and middle-income countries (LMICs) especially in Africa (5, 6).

**Table 1:** FDA-approved ARVs.

Drug class	Mechanism of action	FDA approved drug	Approval year
Nucleoside Reverse Transcriptase Inhibitors (NRTIs)	Inhibit reverse transcriptase (RT) by binding to the active site of the enzyme (RT) leading to a chain termination	Zidovudine	1987
		Didanosine	1991
		Zalcitabine	1992
		Stavudine	1994
		Lamivudine	1995
		Delavirdine	1997
		Abacavir	1998
		Tenofovir Disproxil Fumarate	2001
		Emtricitabine	2003
Non-Nucleoside reverse transcriptase inhibitors (NNRTIs)	Inhibit RT by direct binding and inactivation	Nevirapine	1996
		Efavirenz	1998
		Etravirine	2008
		Rilpivirine	2011
		Nevirapine (XR)	2011
		Doravirine	2018
Protease Inhibitors (PIs)	Inhibit HIV protease (required for catalytic cleavage of viral proproteins for new virion assembly)	Saquinavir	1995
		Indinavir	1996
		Nelfinavir	1997
		Lopinavir/Ritonavir	2000
		Atazanavir	2003
		Fosamprenavir	2003
		Darunavir	2006
Fusion inhibitors (FIs)	Block entry of virus into CD4 receptor expressing cells	Enfuvirtide	2003
CCR5 antagonists	Bind and block the CCR5 receptors on host cells surface preventing entry	Maraviroc	2007
Integrase inhibitors	Inhibit integration of viral cDNA into host cell by integrase enzyme	Raltegravir	2007
		Elvitegravir	2012
		Dolutegravir	2013
		Bictegravir	2018
		Cabotegravir	2021
Attachment inhibitors	Binds gp120 surface protein of HIV preventing interaction with CD4 cell surface receptors	Fostemsavir	2020
Post-attachment inhibitors	Block CD4 receptors on surface of target cells preventing the virus from using them to infect cells	Ibalizumab-uiyk	2018
Capsid inhibitors	Interfere with HIV capsid	Lenacapavir	2022
Pharmacokinetic Enhancers (PE)	Enhances the effectiveness of HIV medicine by inhibit human CYP3A protein increasing plasma concentration of other anti-HIV drugs	Cobicistat	2014

XR**,** extended release.

The guidelines for HIV treatment and monitoring adopted in resource-limited settings are different from those used in high-income countries (HICs) (7). For instance, WHO recommends the use of 1,000 copies/ml as cut-off for virological failure in LMICs setting while a more stringent cutoff of 50 copies/ml is adopted HICs (8). Moreover, some of ART regimens used in Africa consist of first-generation drugs, which have been discontinued in HICs. Triomune™ also known as Viramune™ is a triple drug combination, consisting of Stavudine (d4T), Lamivudine (3TC), and Nevirapine (NVP), formulated as a fixed dose combination (FDC) (9). This FDC is widely used in LMICs due to its low production cost but it has been associated with toxicity (10, 11). While Triomune™ is affordable, the toxicity associated with it adversely affect adherence and its use has been restricted in several resource-rich countries (12). Atripla™, the Gold standard drug since 2006 produced by Gilead Sciences Inc, Foster City, CA, USA and Bristol-Myers Squibb, New York City, NY, USA) contain Efavirenz, Emtricitabine, and Tenofovir disoproxil fumarate (13) was discontinued in Global North (14) due to psychiatric adverse events of Efavirenz (14, 15). The success of integrase inhibitor-based FDCs such as Stribild, Triumeq and Biktarvy also contributed to discontinuation of Atripla™ in HICs.

Prior to 2019, Tenofovir + Lamivudine (or Emtricitabine) + Efavirenz was the first line regimen used in Ghana (16). Ghana adopted the Dolutegravir (DTG) based ARTs in 2019 and since then, the preferred first line regimen has been Tenofovir (TDF) + Lamivudine (3TC) (or Emtricitabine (FTC)) + Dolutegravir (DTG) (17). The second line ART regimen used in Ghana include Zidovudine **+** Lamivudine (or Emtricitabine) **+** Lopinavir/r (or Atazanavir/r) or Tenofovir + Lamivudine (or Emtricitabine) + Lopinavir/r (or Atazanavir/r). The third line ART regimen includes Darunavir/r + Dolutegravir (or Raltegravir ± 1 or 2 NRTI combinations) (17). The integrase inhibitor, dolutegravir (DTG), which was considered “a wonder drug” has reduced efficacy (18) especially in presence of pre-treatment drug resistance mutations to reverse transcriptase inhibitors (19). The development of resistance to DTG underscores the need to screen for pre-treatment drug resistance prior to initiation of DTG-based ART. However, in LMICs, particularly sub-Saharan Africa, where access to drug resistance testing is limited, this is rarely done.

The pipeline for HIV drugs development has yielded novel therapeutics which are more tolerable and highly efficacious. However, these newer drugs are mainly available in HICs. Africa, the region with majority of PLWH is often last on the list to receive these effective drugs. In addition to the high burden, the continent is also plagued with diverse HIV subtypes and recombinant forms which spread and sustain epidemics in different sub-regions of the continent. Thus, the need to make available newer and more effective ARVs in Africa is more urgent than ever.

## Challenges of HIV management in Africa

HIV prevention and treatment in Africa trails behind HICs due to the prohibitive cost, unavailability of newer potent antivirals, limited diagnostic capacity and high burden of co-morbidities such as tuberculosis among PLWH (20, 21). Some of the reagents for HIV monitoring require cold storage and shipment, which is limited in developing countries. Regular monitoring of viral load remains the most effective method to assess patient's response to ART, but this is not routinely performed in most African countries (22). The WHO recommends viral load testing after 6 months of ART (23, 24). In the event of persistent virologic failure despite optimal adherence, the clinician may request for a drug resistance test to determine if viral failure is driven by drug resistance mutations. In cases where resistance is detected, the recommendation is to switch the patient to a different regimen. Transmitted drug resistance mutations against commonly used ARTs have been reported in Ghana, however, drug resistance testing is not routinely conducted before ART initiation (25, 26). Clinicians therefore switch treatment without empirical evidence of drug resistance, which may exacerbate the problem of drug resistance to second and third line drugs (27).

## It is cost—effective to bring newer, more effective drugs to Africa now

Despite the high cost of newer drugs, they have many benefits (28). For instance, they are more effective, less toxic and can help PLWH achieve viral suppression, which significantly reduces the risk and long-term costs of transmitting the virus (29). Thus, PLWH can stay healthier for longer periods of time, eliminating the need for hospitalization and costly medical interventions. Consequently, this would help to reduce HIV incidence, improve life-years and quality-adjusted life-years (QALYs) (30). The newer drugs would also help to prevent opportunistic infections since prolonged viral suppression is associated with immune recovery (31). Achieving viral remission or undetectable viral load is particularly important for PLWH since it affords them a healthy life, allowing them to continue working to improve their economic status.

With a rise in global migration, there is an impending risk of transmission of HIV from high endemic regions in Africa to HICs. Therefore, for complete eradication, the sustained incidence in LMICs must be dealt with by making available newer, more effective drugs in these regions where they are needed most (32, 33). Overall, investing in more effective newer HIV drugs is not only crucial to improve health outcomes, but also helps to reduce the risk of transmission (34).

## Targets of current available antiretroviral therapy

There are ten classes of drugs available for the treatment of HIV, which target different stages of the virus replication cycle. These classes include Nucleoside/nucleotide reverse transcriptase inhibitors (NRTIs), non-nucleoside reverse transcriptase inhibitors (NNRTIs), protease inhibitors (PIs), Integrase strand transfer inhibitors (INSTIs), fusion inhibitors, CCR5 antagonists, attachment inhibitors, post-attachment inhibitors, capsid inhibitors and pharmacokinetic enhancers (35–37). These classes include 24 unique compounds, and 23 combination compounds approved by the US-FDA for treatment of HIV infection (37). Mode of action and drugs in each class are shown in Table 1.

A strategy to enhance treatment success involves simultaneous use of drugs from different classes in form of a combination therapy, which are currently the standard of care for antiretroviral therapy. Combination therapies were first introduction with the development of protease inhibitor, Saquinavir and has evolved over time culminating in the development of fixed dose combinations (FDCs) (38). Combining multiple drugs from different classes helps to reduce development of drug resistance while enhancing treatment success.

## Africa is left to use the “old” drugs

The mainstays of antiretrovirals (ARVs) for management of HIV have largely consisted of a handful of drug classes for many years. Nucleoside reverse transcriptase inhibitors (NRTIs) were the first FDA-approved HIV drugs (39). Subsequently more classes of ARVs targeting different HIV replication cycle stages were discovered and included in treatment regimen (40). After a prolonged use of these drugs, their efficacy reduces due to development of resistance. In HICs, drugs that lose efficacy are replaced by newer, more effective drugs to achieve and maintain viral suppression. However, the “old” drugs, with reduced efficacy are still used in LMICs since they are affordable and can be produced generically. One of the newly FDA-approved drugs, Lenacapavir, has demonstrated high efficacy when used twice-yearly as a pre-exposure prophylaxis (PrEP) (41), but is only available in HICs. The high cost of Lenacapavir is a major obstacle to its availability in LMICs where it is needed most.

## Suboptimal viral load suppression outcomes in the era of “old” drugs

The use of less effective ART in resource limited settings has negative ramifications for realization of the UNAIDS ambitious goals of achieving 95% viral suppression among PLWH on ART (42, 43). Viral suppression rate of 31% was reported in Ghana in 2019 (44). Four years after the introduction of DTG-based regimen in Ghana, viral suppression rate appears to have improved slightly to 40% in 2023 (45). Numerous factors are likely to contribute to this low rates of viral load suppression including non-adherence to treatment, stigma, drug resistance, and low drug efficacy (46). The continued use of less effective drugs in resource-limited setting contributes significantly to poor virological outcomes.

In Ethiopia, an incidence of 30% virological failure was reported in 2015 (47). Similar high incidence of virologic failures have been reported in Uganda (34%) (48), Mozambique and Uganda (29%) (49), Nigeria (33.3%) (50). Data on viral load suppression rate in Ghana and other sub-Saharan African countries suggest poor virological outcome among PLWH on ART underscoring the need to make newer and more effective drugs in LMICS.

## Newer drugs are more effective but unavailable in Africa

Newer HIV drugs receiving FDA approval are more efficacious and if used appropriately can improve viral suppression rates. However, most of these drugs are not available in resource-limited setting due to their high cost. Some of the newer drugs were based on repurposing available drugs and new combination drugs. In 2018, a novel drug, Ibalizumab-uiyk was approved (51). The approval of ibalizumab-uiyk was followed by an NNRTI, Doravirine and a novel attachment inhibitor, Fostemsavir in 2020 (52). Fostemsavir is prescribed for patients with multi-drug resistant HIV who fail treatment due to resistance or intolerance (53). Lenacapavir, is a newly approved capsid inhibitor, which demonstrated 100% transmission prevention efficacy and superiority compared to background HIV incidence in cis-gender women (54). While the phase III clinical trial (PURPOSE 1) (NCT04994509) (54) was conducted in Uganda and South Africa leading the US-FDA approval, this drug is not available for use in most African countries. In addition to the high efficacy, Lenacapavir is administered twice yearly as a long acting injectable eliminating the need for daily pills, hence improving adherence. Improved adherence enhances treatment outcome (55). Making some of these newer drugs available to LMICs has long been shown to be a cost-effective approach in the treatment and transmission prevention of HIV/AIDS (56–58). There have also been expansions in approved indications for previously available ARVs (Table 2), which offers newer options for certain patient populations and indications (59). However, despite their improved efficacy and other advantages associated with newer drugs, they are largely only available to patients in HICs. For example, between 2010 and 2024, a total of 35 ARV entities have received FDA approval but only 3 of these (Nevirapine, Raltegravir and Dolutegravir) are available for use in Ghana (17).

**Table 2:** List of new-indication approvals.

Generic name	Mechanism of action	New indication	Approval year
Darunavir	PI	Pregnant women with HIV	2018
Emtricitabine/tenofovir disoproxil fumarate	NNRTI/NRTI	PrEP in adolescents	2018
Doravirine	NNRTI	Replace the current ARV regimen in adult patients virologically suppressed (VL < 50 copies/ml)	2019
Emtricitabine/tenofovir/alafenamide	NRTI/NRTI	HIV PrEP	2019
Dolutegravir	INSTI	Paediatric patients	2020

## Two standards of care for HIV

While PLWH in Ghana and most parts of Africa continue to take three drug combination regimen, more effective single pill regimens are available in HICs. In the USA and United Kingdom, a single pill, Biktarvy composed of Bictegravir, Emtricitabine, and tenofovir alafenamide (BIC/FTC/TAF) or Triumeq made up of dolutegravir/abacavir/lamivudine are used as first line regimen (60, 61). These first line FDCs are efficacious and improve virologic outcome due to enhanced adherence. Owing to these differences in the standard of care for PLWH in HICs and LMICs, while HIV has been transformed into a chronic manageable disease in the HICs, LMICs who do not have access to new effective drugs, continue to bear the brunt of the epidemics a situation that has been highlighted previously (7, 62).

**Figure 1:**
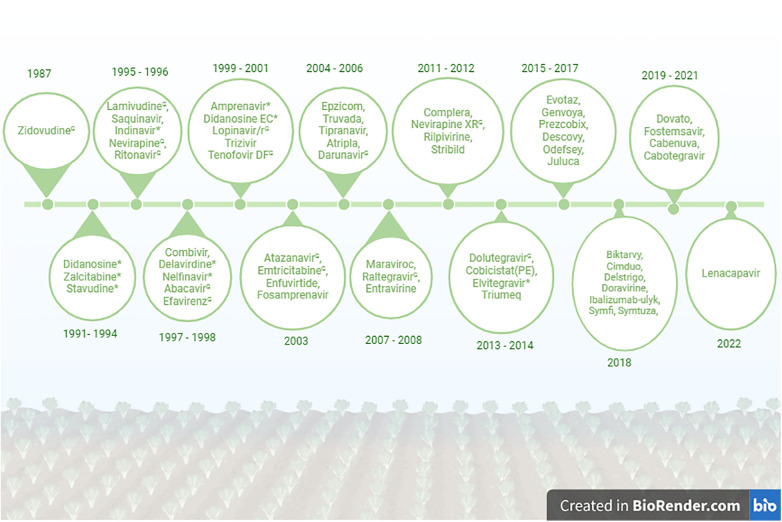
List of FDA approved HIV drugs from 1987 to 2022. *No longer recommended for use in the United States. ^G^Currently used in Ghana. Created in BioRender: BioRender.com/j10o944.

LMICs continue to use less effective and toxic ARVs. Didanosine, Zalcitabine, Stavudine and Elvitegravir are currently not prescribed for treatment of PLWH in HICs continue to be used in Africa (63, 64). Dolutegravir-based treatment had been available and recommended by the World Health Organization (WHO) since 2013, but most African countries were only able to include this drug into their treatment regimen from 2019 (65–67). If this trend is not reversed, most LMICs will miss the United Nations Joint Programme on HIV/AIDS (UNAIDS) 95–95–95 target by 2030. A comprehensive list of FDA approved ARV drugs from 1987 to 2022 is shown in the Figure 1.

## Time to lower the 1,000 copies/ml threshold

Africa has adopted viral suppression threshold of <1,000 copies/ml set by WHO (68). On the contrary, HICs implement use a more stringent criteria of <50 copies/ml for viral suppression. The differences in HIV treatment and monitoring criteria raises the question of double standards (7). The threshold of 1,000 copies/ml for HIV viral load has been used for many years as a key marker to monitor the effectiveness of antiretroviral therapy (ART) among PLWH in Africa (69, 70). This criterion has resulted in a categorization of PLWH with viral loads between 200 and 999 copies/ml as low-level viraemia. These individuals can potentially develop drug resistance yet not much attention is given to them since they are considered low level viremia (71, 72). In a Swedish cohort, low-level viremia in the range of 200–999 copies/ml was associated with virological failure and higher rates of mortality (73). Given the success achieved in HICs and the risks associated with persons classified as low-level viraemia (200–999 copies/ml), justifies the need to emphasize the call to lower viral load suppression threshold. A lower viral load threshold would help to enhance management, curb the risk of developing drug resistance and transmission (74). Even though the WHO reports that individuals with viral loads less than 1,000 copies/ml are less likely to spread the virus, the UNAIDS data showed that people with less than 200 copies/ml cannot transmit the virus. Thus, the need to consider lower viral load suppression threshold policy. Previously, the detection limit for assays used for viral load quantification constituted a formidable barrier to lowering viral load suppression threshold. However, since the introduction of highly sensitive PCR assays detection limits is no longer a challenge to lowering suppression threshold.

## Addressing drug pricing and policy barriers

Access to affordable and quality generic HIV-drugs has increased ART coverage from 610 000 in to 2000 to 29.8 million in 2022 (75). However, intellectual property (IP) protections, including patents to innovator drugs by developers and pharmaceutical companies remains a key barrier in driving down the prices of HIV-drugs for LMICs (76). Patent pools, which grant non-exclusive licenses for intellectual property, is one way that can improve access to new medicines by enabling third-party development (77). Lessons from the COVID-19 pandemic, where intellectual property licensing via bilateral agreements and the Medicines Patent Pool enabled access to new therapeutics in LMICs could be applied in development of newer HIV drugs (78). Initiating this kind of licensing early in the research and development life cycle could facilitate rapid development of generic versions of innovative drug in LMICs.

## Recommendations to improve availability of new drugs

We propose the following recommendations at the government and stakeholder levels to facilitate the prompt availability of new HIV drugs for PLWH in Africa:

(a) Government policy

Governments should implement policies to simplify and harmonize regulatory approvals for new HIV drugs, tailored to local contexts. For example, the Medicines Control Authority of Zimbabwe (MCAZ) successfully expedited the approval of long-acting injectable cabotegravir (CAB-LA) by leveraging prior approval from the US FDA (79). In addition, government policies should support locally driven clinical trials to generate the data necessary for informed regulatory approvals in the region. Moreover, African governments must allocate sufficient funding and invest in infrastructure development, including robust supply.

(b) Stakeholder Level

Enhancing the development, distribution, and accessibility of HIV drugs requires collaboration among key stakeholders, including pharmaceutical companies, academic institutions, regulatory bodies, healthcare providers, and patient advocacy groups. Coordinated efforts between governments, NGOs, and industry partners are essential to accelerate drug development and production. Engaging communities and patient advocacy groups ensures that new HIV drugs address the needs and preferences of those most affected, promoting equity, acceptance, and better health outcomes.

## Conclusion

Enormous progress in ART access has been achieved worldwide. However, in LMICs, significant challenges remain, particularly regarding the standard of care, diagnosis, and quality of drugs provided to PLWH. While in HICs, HIV/AIDS has been transformed into a chronic but manageable condition, the same cannot be said about LMICs, which still grapple with the challenges of accessing effective drugs. We may not achieve the Joint United Nations Programme on HIV/AIDS (UNAIDS) target of 95–95–95 by 2025 and may ultimately miss the opportunity to end HIV/AIDS as an epidemic by 2030 if newer, more effective, and well-tolerated ARTs are not made available in Ghana and the rest of Africa. This will be beneficial in the long term by helping to suppress the virus and reduce the population-wide costs of HIV control and AIDS eradication. Only when a significant proportion of PLWH achieve viral suppression will transmission end. That is essential to defeat the pandemic.
